# William Charles Dement

**DOI:** 10.5935/1984-0063.20200065

**Published:** 2020

**Authors:** Monica L. Andersen, Sergio Tufik

**Affiliations:** Departamento de Psicobiologia, Universidade Federal de São Paulo, São Paulo SP, Brazil.

William Charles Dement was born in Wenatchee, Washington, USA, on 29^th^ July 1928 and died on 17^th^ June 2020. He was a pioneer in sleep studies as he created the fields of sleep research and sleep medicine. He started studying sleep in 1950s at the University of Chicago. Together with Nathaniel Kleitman and Eugene Aserinsky, he discovered and described rapid eye movement (REM) sleep - the phase of sleep in which dreams are most likely to occur. Then, during his internship at Mount Sinai Hospital in New York, he started a sleep laboratory in his Manhattan apartment, where he used to study the impact of dream deprivation on REM sleep-deprived dancers. In the 1960s he moved to Stanford where he became a legendary professor at Stanford School of Medicine. His course Sleep and Dreams has been attended by around 20,000 undergraduate students. His contribution to the sleep field was tremendous with the foundation of the world’s first sleep disorders center in 1970, and the first professional organization for sleep research in 1975, now the American Academy of Sleep Medicine, of which he was president for 12 years. In 1978, together with his colleague, Christian Guilleminault, he founded the journal Sleep^1^.

Over the following decades he made amazing contributions to Sleep Science, including the elucidation of the phases of the human sleep cycle and identification of the physiological basis of dreams. For him, the world dangerously undervalued the importance of sleep and the negative effects of sleep deprivation , and his mission was to educate and alert the world about the consequences of a sleep deprived society. With Christian Guilleminault, he recognized one of the most common chronic sleep disorders - sleep apnea, and began work on the development of treatments. Sleep research was advanced by other significant developments made by Deent - including polysomnography, which provided more parameters to diagnose sleep disorders, including blood oxygen levels, breathing and heart rates, leg movements and snoring. It became the gold standard method to evaluate sleep and its disorders, playing an extremely important in Sleep Medicine. In the 1990s, Dement was Chairman of the National Commission on Sleep Disorders Research, which gave him the opportunity to increase awareness of sleep conditions and resulted in the establishment of the National Center on Sleep Disorders Research^2,3^.

*Some Must Watch While Some Must Sleep* and *The Promise of Sleep* are two of several books written by Dement. Based on his class Sleep and Dreams at University of Stanford, he wrote the book *Dement´s Sleep and Dreams*. As an honored professor of the Institution, the University of Stanford created a tribute video for his 80^th^ birthday, marking 45 years’ service to Stanford. Following his death, researchers from all over the world expressed their feelings about this giant in Sleep Medicine, as can be observed by the many articles and news items that have been published.

His legacy involves not only books, discoveries, and research in the sleep field, he also dedicated his time to make society understand the negative effects of sleep deprivation, which helped to improve health through sleep, and saved many lives through diagnosis and treatment.
